# The constitutive level of vascular endothelial growth factor (VEGF) is more important than hypoxia-induced VEGF up-regulation in the angiogenesis of human melanoma xenografts

**DOI:** 10.1054/bjoc.2000.1173

**Published:** 2000-04-03

**Authors:** T Danielsen, E K Rofstad

**Affiliations:** Department of Biophysics, The Norwegian Radium Hospital, Montebello, Oslo, N-0310, Norway

**Keywords:** angiogenesis, hypoxia, immunohistochemistry, VEGF

## Abstract

Angiogenesis of tumours might develop as a result of environmental conditions, such as hypoxia, and/or as a result of genetic alterations specific for tumour cells. The relative contributions of these mechanisms were investigated by comparing the in vivo expression of vascular endothelial growth factor (VEGF) and basic fibroblast growth factor (bFGF) to the hypoxic fraction, the angiogenic potential and the vascular density of four human melanoma lines (A-07, D-12, R-18, U-25) grown intradermally in Balb/c *nu/nu* mice. VEGF expression, bFGF expression and expression of pimonidazole, a marker of hypoxic cells, were investigated by immunohistochemistry. An association between high VEGF and bFGF expression and high angiogenic potential was detected, suggesting an important role for VEGF/bFGF in the angiogenesis of melanomas. High VEGF/bFGF expression was also related to low hypoxic fraction and high vascular density. Thus, the constitutive, genetically determined level of VEGF was probably more important than hypoxia-induced upregulation in the angiogenesis of the melanoma xenografts. © 2000 Cancer Research Campaign


*British Journal of Cancer* (2000) **82**(9), 1528–1534
© 2000 Cancer Research Campaign
DOI: 10.1054/ bjoc.2000.1173, available online at http://www.idealibrary.com on IDEAL®

### The constitutive level of vascular endothelial growth factor (VEGF) is more important than hypoxia-induced VEGF up-regulation in the angiogenesis of human melanoma xenografts

**T Danielsen and EK Rofstad**

Department of Biophysics, The Norwegian Radium Hospital, Montebello, N-0310 Oslo, Norway

**Summary** Angiogenesis of tumours might develop as a result of environmental conditions, such as hypoxia, and/or as a result of genetic alterations specific for tumour cells. The relative contributions of these mechanisms were investigated by comparing the in vivo expression of vascular endothelial growth factor (VEGF) and basic fibroblast growth factor (bFGF) to the hypoxic fraction, the angiogenic potential and the vascular density of four human melanoma lines (A-07, D-12, R-18, U-25) grown intradermally in Balb/c *nu/nu* mice. VEGF expression, bFGF expression and expression of pimonidazole, a marker of hypoxic cells, were investigated by immunohistochemistry. An association between high VEGF and bFGF expression and high angiogenic potential was detected, suggesting an important role for VEGF/bFGF in the angiogenesis of melanomas. High VEGF/bFGF expression was also related to low hypoxic fraction and high vascular density. Thus, the constitutive, genetically determined level of VEGF was probably more important than hypoxia-induced upregulation in the angiogenesis of the melanoma xenografts. © 2000 Cancer Research Campaign

**Keywords**: angiogenesis; hypoxia; immunohistochemistry; VEGF

Vascular endothelial growth factor (VEGF) is probably the single angiogenic inducer that contributes the most to the angiogenesis of human tumours (Kim et al, 1993; Pötgens et al, 1995; Claffey et al, 1996). Basic fibroblast growth factor (bFGF) might promote tumour angiogenesis by interacting with VEGF (Goto et al, 1993; Seghezzi et al, 1998). VEGF is up-regulated when cells are exposed to hypoxia in vitro and nearby necrosis in vivo (Shweiki et al, 1992; Hlatky et al, 1994). Other more direct studies have also suggested that induction of VEGF by hypoxia is an important signal for tumour angiogenesis. Shweiki et al (1995) have demonstrated up-regulation of VEGF in the centrally located, hypoxic cells of spheroids grown in vitro, which was followed by vascularization and VEGF down-regulation when the spheroids were transplanted in vivo. Moreover, hypoxia-induced upregulation of VEGF in human tumour cells in vitro can increase tumour angiogenesis when the cells are grown as xenografts in vivo (Rofstad and Danielsen, 1998). The observation that hypoxia stimulates angiogenesis, together with the fact that VEGF is up-regulated in normal cells by hypoxia (Shweiki et al, 1992; Hlatky et al, 1994), suggest tumour angiogenesis to be an inherent physiological process, i.e. an example of tissue signalling its environment to provide increased vascularization (D'Amore and Shima, 1996). If this is the main mechanism of angiogenesis in tumours, one would expect strong VEGF expression in relatively hypoxic tumours, while well-oxygenated tumours would show little VEGF expression. A correlation between high VEGF protein expression and hypoxia was indeed seen in rectal carcinoma (Mattern et al, 1996).

*Received 13 August 1999*
*Revised 4 January 2000*
*Accepted 9 January 2000*

*Correspondence to:* EK Rofstad

Although different VEGF levels in tumours can originate as a result of hypoxia, VEGF secretion and mRNA levels do also differ significantly between tumour cells of the same histological origin grown under normoxic conditions in vitro (Westphal et al, 1997; Rofstad and Danielsen, 1998). Thus, it seems likely that genetic changes during tumour progression modulate the VEGF expression of the tumour cells. An increase in VEGF expression can be an important step in the tumorigenic process, as it promotes angiogenesis and thereby tumour growth (D'Amore and Shima, 1996). If the constitutive, genetically determined level of VEGF is an important factor in tumour angiogenesis, a high vascular density would be expected in tumours with a high VEGF expression. A correlation between high vascular density in vascular 'hot spots' of the tumours and high VEGF expression has been observed in different histological types of clinical cancer specimens (Guidi et al, 1995; Takahashi et al, 1995; Obermair et al, 1997). Moreover, transfection of VEGF into human cancer cells has been shown to increase the vascular density of tumour xenografts (Claffey et al, 1996).

The angiogenesis of tumours can probably be regulated both by normal physiological mechanisms and by genetic mechanisms specific for tumour cells. Thus, Mazure et al (1996) have found hypoxia and oncogenic transformation to act synergistically in the modulation of VEGF expression in rodent fibroblasts. However, the relative importance of hypoxia-induced and genetically determined VEGF expression in the angiogenesis of human tumours has not been studied. Therefore, we wanted to investigate the possibility that VEGF-induced angiogenesis is mediated mainly by one of these mechanisms in melanomas. Four human melanoma xenografts (A-07, D-12, R-18 and U-25) that are wild-type for *TP53* were used as a model system, in which VEGF and bFGF expression in vivo were compared to the hypoxic fraction, the angiogenic potential and the vascular density.

### MATERIALS AND METHODS

### Mice and tumour lines

Adult Balb/c *nu*/*nu* mice, bred at our research institute, were used as host animals for xenografted tumours. The mice were maintained under specific pathogen-free conditions at constant temperature (24–26°C) and humidity (30–50%). Sterilized food and tap water were given ad libitum.

The experiments were performed using four human melanoma cell lines (A-07, D-12, R-18, U-25) (Rofstad, 1994). Xenografted tumours were initiated from exponentially growing monolayer cultures in passages 75–100. Monolayer cells, cultured in RPMI-1640 medium (25 mM HEPES and L-glutamin) supplemented with 13% fetal bovine serum (FBS), 250 mg $l^{-1}$ penicillin and 50 mg $l^{-1}$ streptomycin, were detached by trypsinization (treatment with 0.05% trypsin/0.02% EDTA solution at 37°C for 2 min). Approximately $3.5 \times 10^5$ cells in 10 μl of $Ca^{2+}$- and $Mg^{2+}$-free Hanks' balanced salt solution (HBSS) were inoculated intradermally in the flanks of mice by using a 100 μl Hamilton syringe. Tumour volume ($V$) was calculated as $V = \pi/6 \times ab^2$, where $a$ is the longer and $b$ is the shorter of two perpendicular tumour diameters, measured with calipers. Tumours with volumes within the range of 200–400 $mm^3$ were used for experiments. The growth and histological appearance of the tumours have been described in detail previously (Rofstad, 1994).

### Immunohistochemical analysis of VEGF and bFGF expression

Immunohistochemical staining of tumour tissue was performed using the avidin–biotin immunoperoxidase method. Tumours were fixed in 4% phosphate-buffered paraformaldehyde and embedded in paraffin casts (VEGF staining), or dropped into liquid $N_2$ immediately after excision and kept at –80°C until staining (bFGF). The paraffin sections (5 μm thick) were treated with 0.6% hydrogen peroxide ($H_2O_2$) to block endogenous peroxidase and all sections were blocked with normal goat serum before incubating with the primary antibody (30-min incubation). The primary antibodies were the rabbit polyclonal antibodies VEGF (A-20) used at 5.0 and 10.0 μg $ml^{-1}$ (Santa Cruz Biotechnology, Santa Cruz, CA, USA) and bFGF (147) used at 2.5 μg $ml^{-1}$ (Santa Cruz Biotechnology). Controls included omission of primary antibody, incubation with normal rabbit immunoglobulin or normal rabbit serum and incubation with blocking peptides against VEGF and bFGF (Santa Cruz Biotechnology) before staining. The sections were counterstained with haematoxylin. Optimal antibody concentrations were found in preliminary titration experiments. The immunostaining for VEGF was performed in two batches, staining two tumours from each tumour line in the first and three tumours from each tumour line in the second batch. Care was taken in order to achieve the same experimental conditions for all tumour sections.

After staining, all sections were studied under light microscope for evaluation of protein localization (intracellular vs extracellular) and intra-tumour and inter-tumour heterogeneity in protein expression. In order to compare the VEGF staining of the four melanoma lines, pictures from the sections (approximately 600 × 600 μm) were displayed on a monitor. The pictures were recorded by the image processing system KS300 (Kontron Elektronik, Munich, Germany), connected to the microscope through a CCD-camera. Equal light conditions and image processing parameters were used for all tumour lines. Five tumours of each line and two pictures of each tumour, preferably from different sections, were subjected to analysis. The VEGF staining of the pictures was evaluated by two slightly different methods. In the first method, four pictures, one from each melanoma line, were displayed on the monitor, compared according to staining intensity and ranked. This procedure was performed for all pictures, and a final ranking was made based on the total amount of data. In the second method, each picture was evaluated separately and given a number according to staining intensity (1, weak staining; 2, intermediate staining; 3, strong staining). The resulting data from this semi-quantitative evaluation were thereafter analysed by statistical methods in order to reveal statistically significant differences in staining intensity between the tumour lines.

### Measurements of metabolic hypoxia

The hypoxia marker pimonidazole was used to measure fraction of metabolically hypoxic cells, i.e. cells that have sufficiently low oxygen tension to metabolize and bind pimonidazole (Raleigh et al, 1998). Pimonidazole is a nitroimidazole, which is selectively metabolized by hypoxic cells to a form that binds covalently to cellular macromolecules. The binding of nitroimidazoles shows a similar dependence on the concentration of oxygen as cellular radiosensitivity.

Mice were given an intraperitoneal injection of pimonidazole in doses of 30 μg $g^{-1}$ body weight approximately 4 h before the mice were killed. The pimonidazole-marked tumours were excised, fixed in 4% phosphate-buffered paraformaldehyde and cut into four parts before embedding in paraffin casts. The sections were stained with anti-pimonidazole using the avidin–biotin immunoperoxidase method. The paraffin sections (5 μm thick) were treated with 3.0% $H_2O_2$ to block endogenous peroxidase, blocked with normal goat serum and treated with proteinase E (0.1 mg $ml^{-1}$) to digest enzymes prior to primary antibody incubation (overnight incubation). Rabbit polyclonal anti-pimonidazole was a personal gift from Dr JA Raleigh. Controls included omission of primary antibody and incubation with normal rabbit immunoglobulin or normal rabbit serum. The sections were counterstained with haematoxylin. Optimal antibody concentration was found in preliminary titration experiments.

The fraction of a tumour showing pimonidazole staining was found by using the image processing system KS300 (Kontron Elektronik, Munich, Germany). The metabolic hypoxic fraction was defined as the pimonidazole-stained area fraction of the non-necrotic tumour area. The area fraction of each section that contained non-necrotic tumour tissue was found by subtracting the necrotic area fraction from the total area. For each melanoma line, 16–25 tumours were analysed, using 2–4 sections from different parts of each tumour and up to 100 randomly selected fields of view.

### Statistical analysis

Statistical comparisons of data were performed by one-way analysis of variance (ANOVA) under conditions of normality and equal variance and otherwise by the Kruskal–Wallis one-way ANOVA on ranks. Student–Newman–Keul's test was thereafter used to identify groups that differed from the others. A significance criterion of $P < 0.05$ and two-sided tests were used. The

© 2000 Cancer Research Campaign

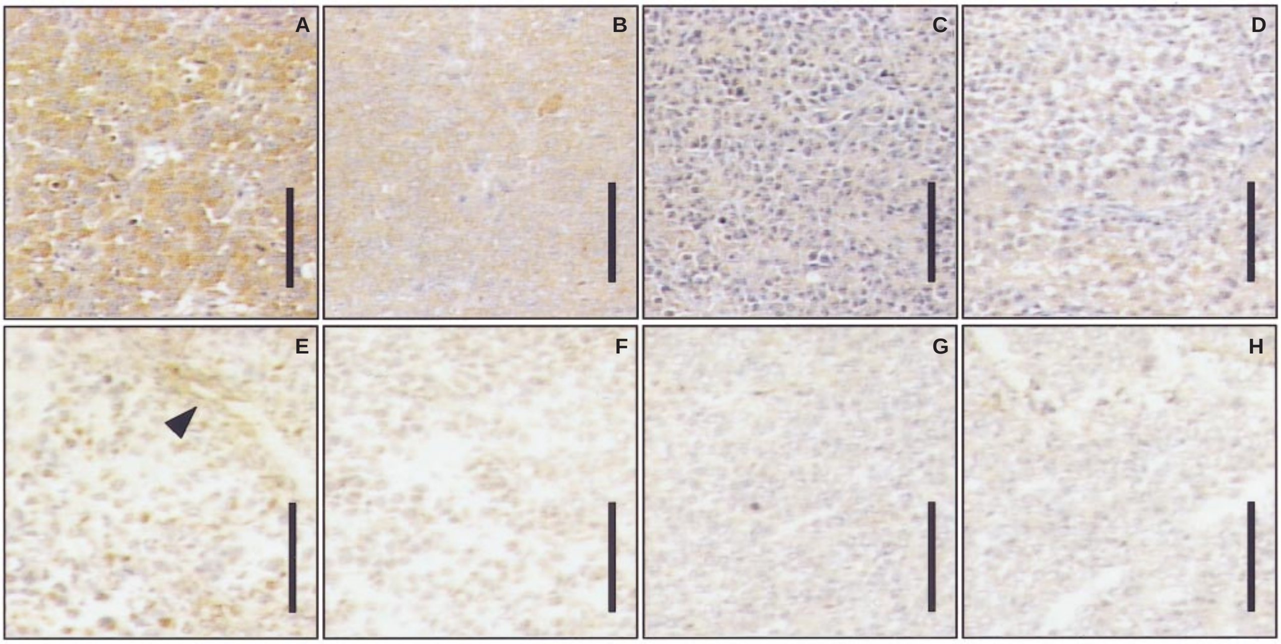

**Figure 1** Immunohistochemical preparations of the human melanoma xenografts A-07 (**A** and **E**), D-12 (**B** and **F**), R-18 (**C** and **G**) and U-25 (**D** and **H**). Paraformaldehyde-fixed sections were stained with anti-VEGF (**A–D**)(5 μg $ml^{-1}$), and frozen sections were stained with anti-bFGF (**E**–**H**) (2.5 μg $ml^{-1}$), using the avidin–biotin immunoperoxidase method. All sections were counterstained with haematoxylin. bFGF staining around a blood vessel is shown in panel **E** (arrowhead). Scale bars, 100 μm

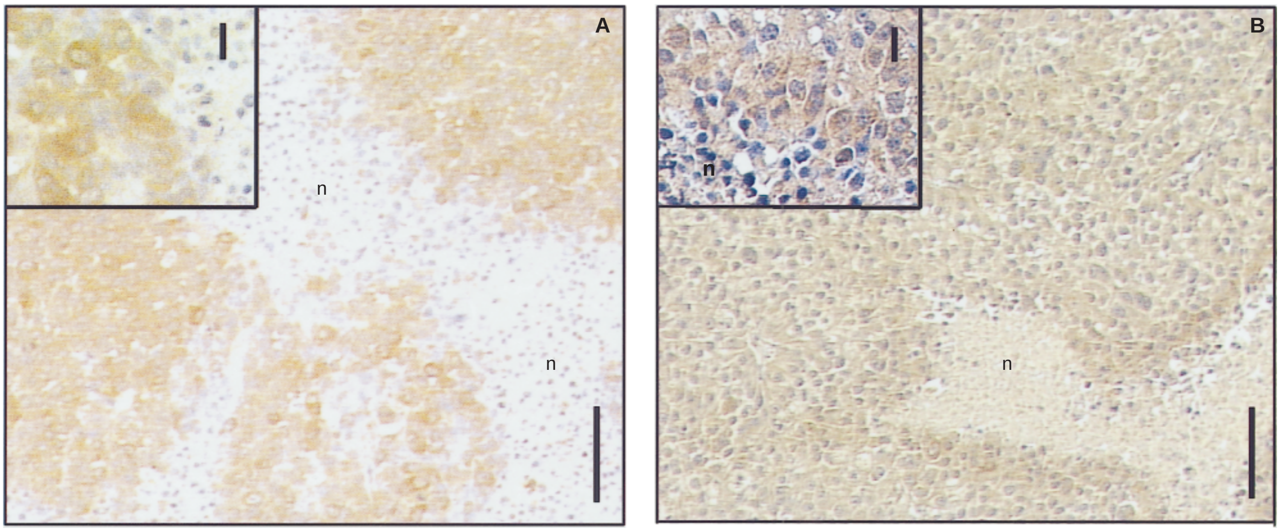

**Figure 2** Immunohistochemical preparations of the human melanoma xenografts D-12 (**A**) and U-25 (**B**). Paraformaldehyde-fixed sections were stained with anti-VEGF (10 μg $ml^{-1}$) by the avidin–biotin immunoperoxidase method and counterstained with haematoxylin. Scale bars, 100 μm and 20 μm (high magnifications); n, necrosis statistical analysis was performed using Sigmastat statistical software (Jandel, Erkrath, Germany).

### RESULTS

Immunohistochemical staining of tumour sections with an antibody specific for VEGF revealed VEGF expression in all tumours of all four melanoma lines. The positive staining seemed to be localized mainly in the cytoplasm of the cells (Figure 1, A–D). Within a tumour, the staining was relatively homogenous, and only a very few negative cells were observed. VEGF was slightly up-regulated nearby necrosis in some areas of the tumours (Figure 2). This up-regulation was most clearly seen in the U-25 line, where cells adjacent to necrotic areas often showed slightly higher VEGF expression than other cells (Figure 2B). The D-12 line also showed up-regulation of VEGF close to necrosis, however, stronger staining was only seen in some of the cells, and cells with high VEGF expression were distributed over a larger distance away from the necrotic area than what was seen in U-25 (Figure 2A). The two lines A-07 and R-18 showed little or no necrosis.

*© 2000 Cancer Research Campaign*

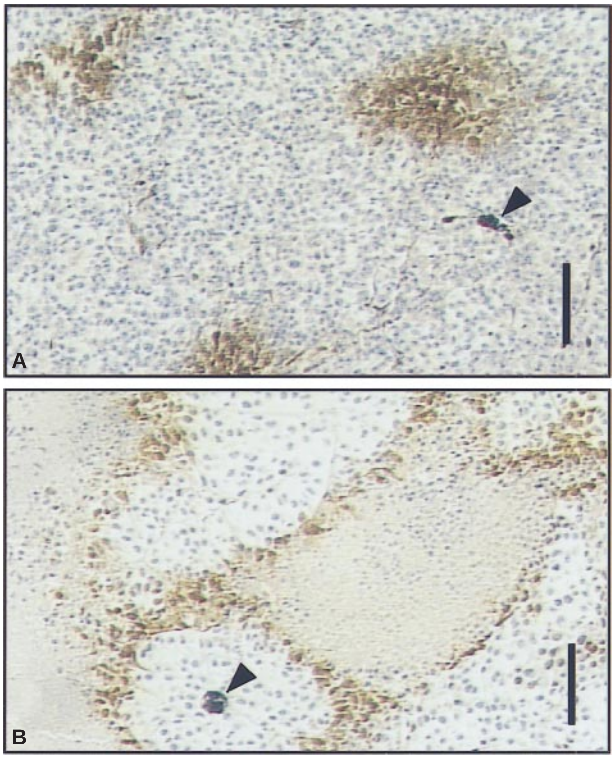

**Figure 3** Immunohistochemical preparations of the human melanoma xenografts R-18 (**A**) and U-25 (**B**) stained with anti-pimonidazole using the avidin–biotin immunoperoxidase method and counterstained with haematoxylin. Scale bars, 100 μm; n, necrosis; arrowheads, blood vessels

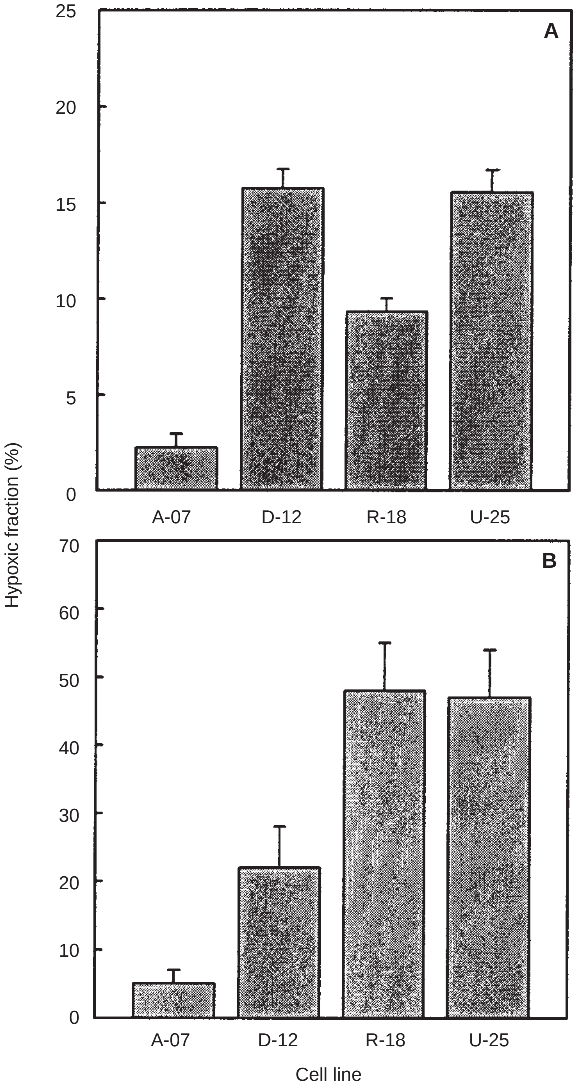

**Figure 4** Hypoxic fractions of the human melanoma xenografts A-07, D-12, R-18 and U-25. (**A**) Metabolic hypoxic fractions obtained by staining tumour sections with the hypoxia marker pimonidazole and measuring the stained area fraction of the non-necrotic tissue. Columns and bars represent the mean ± s.e.m. of 16–25 tumours. (**B**) Radiobiological hypoxic fractions obtained by the local tumour control assay (Rofstad and Måseide, 1998)

The VEGF staining was found to be substantially stronger for A-07 than for the other three lines when groups of four pictures were compared (i.e. A-07 was ranked number one in almost all groups). Moreover, the staining of D-12 was slightly stronger than that of R-18 and U-25 by this method (i.e. D-12 was ranked number two more often than either R-18 or U-25). Significantly higher VEGF staining of A-07 than of the other three lines was also detected when pictures were evaluated separately ($P < 0.05$). No differences in staining intensities between D-12, R-18 and U-25 were detected by this method ($P > 0.05$). The staining was performed in two batches. No differences in the evaluation results were found when the two groups were evaluated separately.

Positive bFGF staining was seen in all four tumour lines by immunohistochemical staining (Figure 1, E–H). The staining of A-07 was mainly localized in intensively stained areas intracellularly. Weaker staining of areas close to blood vessels and extracellularly was also seen in A-07 (Figure 1E). The other three lines showed weaker intracellular staining than A-07, but the staining nearby blood vessels and extracellularly was comparable to that of A-07 (Figure 1, F–H). The bFGF expression of individual tumours was quite homogenous for all the lines, and no negative areas were observed.

Pimonidazole staining of tumour sections was assessed to determine the fraction of metabolically hypoxic cells in the tumour lines. Tumour sections showing positive pimonidazole staining are shown in Figure 3. The well-defined stained areas were seen as blotches in tumour tissue without significant necrosis (Figure 3A), or adjacent to large necrotic areas (Figure 3B). Pimonidazole staining was not directly associated with VEGF staining, as it was seen in certain areas of the tumours only, while VEGF staining was observed all over the tumours, except for some necrotic areas. Pimonidazole staining was not directly associated with VEGF up-regulation either, as it was often detected in areas without necrosis, whereas VEGF up-regulation was only seen close to necrotic areas. Moreover, pimonidazole staining was seen adjacent to all necrotic areas, while VEGF was slightly up-regulated close to some of the necrotic areas only.

The fraction of metabolically hypoxic cells was significantly lower for A-07 than for the three other lines ($P < 0.05$) (Figure 4A). R-18 showed a significantly lower fraction of metabolically hypoxic cells than D-12 and U-25 ($P < 0.05$). The fraction of metabolically hypoxic cells did not differ significantly between D-12 and U-25 ($P > 0.05$).

By comparing the fraction of metabolically hypoxic cells to data published previously (Rofstad and Måseide, 1998) on the fraction of radiobiologically hypoxic cells (Figure 4B), both similarities

 © 2000 Cancer Research Campaign

**Table 1** Angiogenic potential and vascular density of human melanoma xenografts

| Melanoma | Angiogenic potential[a] | Vascular density[b] |
|---|---|---|
| A-07 | 151 ± 10 | 48 ± 4 |
| D-12 | 64 ± 8 | 22 ± 2 |
| R-18 | 48 ± 6 | 20 ± 2 |
| U-25 | 39 ± 5 | 21 ± 4 |

[a]Number of tumour-oriented capillaries at day 7 after intradermal inoculation of $3.5 \times 10^5$ melanoma cells; mean ± s.e. (Danielsen and Rofstad, 1998).
[b]Vessels/area of non-necrotic tissue (no./mm$^2$); mean ± s.e. (Tufto et al, 1998).

and discrepancies between the assays were observed. A-07 showed a significantly lower fraction both of metabolically hypoxic cells and of radiobiologically hypoxic cells than the other three lines ($P < 0.05$). On the other hand, the significant difference between R-18 and U-25 in fraction of metabolically hypoxic cells was not detected when the fraction of radiobiologically hypoxic cells was measured. Moreover, D-12 showed a significantly lower fraction of radiobiologically hypoxic cells than R-18 and U-25 ($P < 0.05$).

Previously published data on the angiogenic potential of the melanoma cells (Danielsen and Rofstad, 1998) and the vascular density of melanoma xenografts (Tufto et al, 1998) are summarized in Table 1. A-07 showed significantly higher angiogenic potential and vascular density than the other three lines ($P < 0.05$). Whereas significant differences in angiogenic potential were seen between all cell lines ($P < 0.05$), there were no significant differences in the vascular density of the lines D-12, R-18 and U-25 ($P > 0.05$).

### DISCUSSION

Four human melanoma lines showing differential expression of angiogenic inducers in vitro (Danielsen and Rofstad, 1998) were used as tumour models in the present study. When assessing the expression of angiogenic inducers in vivo, it is important to use a tumour model in which the expression is not partly originating from host cells. Several normal cell types have been shown to express VEGF and bFGF at normoxic conditions and to up-regulate VEGF expression at hypoxic conditions (Cordon-Cardo et al, 1990; Shweiki et al, 1992; Hlatky et al, 1994). However, as host cells (macrophages, leucocytes, fibroblasts) constitute less than 1% of the total number of cells in the xenografted melanomas (Rofstad, 1994), VEGF and bFGF detected in vivo are most likely of human origin. Furthermore, both VEGF and bFGF seemed to be localized mainly intracellularly by immunohistochemistry, and both inducers were detected by Western blotting of the melanoma cells grown in vitro (Danielsen and Rofstad, 1998). Accordingly, the model system used here was suitable for in vivo investigation of VEGF and bFGF expression.

VEGF expression was found to be considerably higher in the A-07 line than in the three other lines, both by direct comparison of pictures from the four tumour lines and by semi-quantitative analysis of tumour sections. The high VEGF expression of A-07 might partly be a result of bFGF stimulation, since A-07 also showed substantially higher intracellular bFGF expression in vivo than the other melanoma lines. Our present results are in agreement with in vitro data on the melanoma cells, showing the A-07 cell line to secrete significantly more VEGF and to express considerably more bFGF than the other three cell lines, and to be the only cell line secreting detectable amounts of bFGF (Danielsen and Rofstad, 1998).

Angiogenic potential, measured as number of tumour-oriented capillaries, and mean vascular density were significantly higher for A-07 than for the other three lines. Thus, our data suggest an association between VEGF/bFGF expression in vivo and angiogenesis, indicating that VEGF and possibly bFGF are important for the angiogenesis of the melanoma xenografts. The significance of VEGF in the angiogenesis of the melanoma xenografts is underscored by experiments in which mice injected with melanoma cells were treated with anti-VEGF neutralizing antibody, resulting in significant decrease in the angiogenic potential (Rofstad and Danielsen, 1999).

Measurements of the fraction of metabolically hypoxic cells showed that the hypoxic fraction of A-07 was significantly lower than that of D-12, R-18 and U-25. As different assays of tumour hypoxia measure different parameters, the numerical values of the hypoxic fractions of tumours can be assay-dependent. However, results from the radiobiological assay also indicated that the hypoxic fraction of A-07 was lower than that of D-12, R-18 and U-25. Moreover, as vascular density is related to the oxygenation status of a tumour (Mattern et al, 1996; Lyng et al, 1997), the significantly higher vascular density of A-07 than of the other lines is consistent with the results achieved by determining hypoxic fraction. Measurements of the oxygenation status of the melanoma xenografts by the Eppendorf $pO_2$ histograph also resulted in a higher oxygenation status for A-07 than for the other three lines (H Lyng, unpublished results). Taken together, these data strongly suggest that the hypoxic fraction of A-07 is significantly lower than that of D-12, R-18 and U-25.

Our data did not reveal any relationship between high VEGF expression and high hypoxic fraction. On the contrary, high VEGF expression was related to low hypoxic fraction and high vascular density, suggesting a negative correlation between VEGF expression and hypoxic fraction in the melanoma xenografts. Since a positive correlation between VEGF expression and hypoxic fraction would be expected if the VEGF-induced angiogenesis of the melanomas was mainly mediated through hypoxia, we suggest the angiogenesis of the melanoma xenografts to be driven mainly by other factors than hypoxia-induced VEGF. This conclusion is supported by the lack of co-localization between VEGF staining and staining for the hypoxia marker pimonidazole, an observation that has also been reported for clinical specimens (Raleigh et al, 1998). Our conclusion is not in line with the recent suggestion that the mRNA expression of VEGF may be used as a marker for radiobiologic hypoxia (Chiarotto and Hill, 1999).

Specific, genetic properties of the tumour cells are probably decisive of the VEGF expression and thereby the angiogenesis of the melanomas studied here. This conclusion is supported by several observations. Firstly, contrary to the results of Pötgens et al (1995), our data show the pattern of VEGF and bFGF expression to be similar whether the melanoma cells are grown as xenografts in vivo or as monolayer cultures under normoxic conditions in vitro (Danielsen and Rofstad, 1998). The growth conditions in vitro were equal for all cell lines, and differences in VEGF/bFGF expression between the lines should therefore represent genetic differences. Secondly, as the VEGF/bFGF expression of the melanoma lines in vivo is related to the vascular density, it is likely that the constitutive, genetically determined level of VEGF is decisive for the

© 2000 Cancer Research Campaign 

angiogenesis and thus the hypoxic fraction of the melanoma lines, and not the other way round, that hypoxia is decisive for the VEGF level. Thus, the angiogenesis of the melanoma xenografts is probably driven mainly by genetic mechanisms, and to a less extent by hypoxia-induced VEGF. Our results are in accordance with data from Westphal et al (1997), showing high VEGF expression and high mean vascular density to be associated in a study of seven human tumour xenografts. Moreover, the present study is consistent with the view that tumour angiogenesis is a result of both normal physiological processes and genetic properties of the tumour cells (D'Amore and Shima, 1996; Mazure et al, 1996).

When searching for genetic properties important for differences in VEGF expression, *TP53* status is a possible candidate as cells transiently transfected with mutant *TP53* show higher VEGF expression than cells transiently transfected with wild-type *TP53* (Mukhopadhyay et al, 1995). Moreover, expression of VEGF and mutant *TP53*, measured by immunohistochemistry, were related in lung cancer (Fontanini et al, 1995) and in colon cancer (Takahashi et al, 1998). The four melanoma lines used in this study do all express wild-type *TP53* (Danielsen et al, 1999). Thus, the observed difference in VEGF expression between A-07 and the other three lines must be attributed to genetic properties other than *TP53* status.

To summarize, our data suggest that hypoxia is not decisive for VEGF expression and thus angiogenesis of melanomas. The constitutive, genetically determined level of VEGF is probably more important than the hypoxia-induced VEGF in the angiogenesis of this type of tumours. This conclusion is drawn in a tumour model system in which the *TP53* status of all lines is equal, i.e. wild-type *TP53*.

### ACKNOWLEDGEMENTS

We thank Dr JA Raleigh, Radiation Oncology Department, UNC School of Medicine, Chapel Hill, NC, USA for supplying pimonidazole and polyclonal rabbit anti-pimonidazole. The excellent technical assistance of Ms Kristin Nilsen and Mr Olav Groven is gratefully acknowledged. Financial support was received from The Norwegian Cancer Society.

### REFERENCES

Chiarotto JA and Hill RP (1999) A quantitative analysis of the reduction in oxygen levels required to induce up-regulation of vascular endothelial growth factor (VEGF) mRNA in cervical cancer cell lines. *Br J Cancer* **80**: 1518–1524

Claffey KP, Brown LF, del Aguila LF, Tognazzi K, Yeo KT, Manseau EJ and Dvorak HF (1996) Expression of vascular permeability factor/vascular endothelial growth factor by melanoma cells increases tumor growth, angiogenesis, and experimental metastasis. *Cancer Res* **56**: 172–181

Cordon-Cardo C, Vlodavsky I, Haimovitz-Friedman A, Hicklin D and Fuks Z (1990) Expression of basic fibroblast growth factor in normal human tissues. *Lab Invest* **63**: 832–840

D'Amore PA and Shima DT (1996) Tumor angiogenesis: a physiological process or genetically determined? *Cancer Metastasis Rev* **15**: 205–212

Danielsen T and Rofstad EK (1998) VEGF, bFGF and EGF in the angiogenesis of human melanoma xenografts. *Int J Cancer* **76**: 836–841

Danielsen T, Grønlund H, Hvidsten M, Smith-Sørensen B, Børresen-Dale A-L and Rofstad EK (1999) No association between radiosensitivity and *TP53* status, $G_1$ arrest or protein levels of p53, myc, ras or raf in human melanoma lines. *Int J Radiat Biol* **75**: 1149–1160

Fontanini G, Vignati S, Lucchi M, Mussi A, Calcinai A, Boldrini L, Chine S, Silvestri V, Angeletti CA, Basolo F and Bevilacqua G (1997) Neoangiogenesis and p53 protein in lung cancer: their prognostic role and their relation with vascular endothelial growth factor (VEGF) expression. *Br J Cancer* **75**: 1295–1301

Goto F, Goto K, Weindel K and Folkman J (1993) Synergistic effects of vascular endothelial growth factor and basic fibroblast growth factor on the proliferation and cord formation of bovine capillary endothelial cells within collagen gels. *Lab Invest* **69**: 508–517

Guidi AJ, Abu-Jawdeh G, Berse B, Jackman RW, Tognazzi K, Dvorak HF and Brown LF (1995) Vascular permeability factor (vascular endothelial growth factor) expression and angiogenesis in cervical neoplasia. *J Natl Cancer Inst* **87**: 1237–1245

Hlatky L, Tsionou C, Hahnfeldt P and Coleman CN (1994) Mammary fibroblasts may influence breast tumor angiogenesis via hypoxia-induced vascular endothelial growth factor up-regulation and protein expression. *Cancer Res* **54**: 6083–6086

Kim KJ, Li B, Winer J, Armanini M, Gillett N, Phillips HS and Ferrara N (1993) Inhibition of vascular endothelial growth factor-induced angiogenesis suppresses tumour growth in vivo. *Nature* **362**: 841–844

Lyng H, Sundfør K and Rofstad EK (1997) Oxygen tension in human tumours measured with polarographic needle electrodes and its relationship to vascular density, necrosis and hypoxia. *Radiother Oncol* **44**: 163–169

Mattern J, Kallinowski F, Herfarth C and Volm M (1996) Association of resistance-related protein expression with poor vascularization and low levels of oxygen in human rectal cancer. *Int J Cancer* **67**: 20–23

Mazure NM, Chen EY, Yeh P, Laderoute KR and Giaccia AJ (1996) Oncogenic transformation and hypoxia synergistically act to modulate vascular endothelial growth factor expression. *Cancer Res* **56**: 3436–3440

Mukhopadhyay D, Tsiokas L and Sukhatme VP (1995) Wild-type p53 and v-Src exert opposing influences on human vascular endothelial growth factor gene expression. *Cancer Res* **55**: 6161–6165

Obermair A, Kucera E, Mayerhofer K, Speiser P, Seifert M, Czerwenka K, Kaider A, Leodolter S, Kainz C and Zeillinger R (1997) Vascular endothelial growth factor (VEGF) in human breast cancer: correlation with disease-free survival. *Int J Cancer* **74**: 455–458

Pötgens AJ, Lubsen NH, van Altena MC, Schoenmakers JG, Ruiter DJ and de Waal RM (1995) Vascular permeability factor expression influences tumor angiogenesis in human melanoma lines xenografted to nude mice. *Am J Pathol* **146**: 197–209

Raleigh JA, Calkins-Adams DP, Rinker LH, Ballenger CA, Weissler MC, Fowler WC, Novotny DB and Varia MA (1998) Hypoxia and vascular endothelial growth factor expression in human squamous cell carcinomas using pimonidazole as a hypoxia marker. *Cancer Res* **58**: 3765–3768

Rofstad EK (1994) Orthotopic human melanoma xenograft model systems for studies of tumour angiogenesis, pathophysiology, treatment sensitivity and metastatic pattern. *Br J Cancer* **70**: 804–812

Rofstad EK and Danielsen T (1998) Hypoxia-induced angiogenesis and vascular endothelial growth factor secretion in human melanoma. *Br J Cancer* **77**: 897–902

Rofstad EK and Danielsen T (1999) Hypoxia-induced metastasis of human melanoma cells: involvement of vascular endothelial growth factor-mediated angiogenesis. *Br J Cancer* **80**: 1697–1707

Rofstad EK and Måseide K (1998) Fraction of radiobiologically hypoxic cells in human melanoma xenografts measured by using single-cell survival, tumour growth delay and local tumour control as end points. *Br J Cancer* **78**: 893–898

Seghezzi G, Patel S, Ren CJ, Gualandris A, Pintucci G, Robbins ES, Shapiro RL, Galloway AC, Rifkin DB and Mignatti P (1998) Fibroblast growth factor-2 (FGF-2) induces vascular endothelial growth factor (VEGF) expression in the endothelial cells of forming capillaries: an autocrine mechanism contributing to angiogenesis. *J Cell Biol* **141**: 1659–1673

Shweiki D, Itin A, Soffer D and Keshet E (1992) Vascular endothelial growth factor induced by hypoxia may mediate hypoxia-initiated angiogenesis. *Nature* **359**: 843–845

Shweiki D, Neeman M, Itin A and Keshet E (1995) Induction of vascular endothelial growth factor expression by hypoxia and by glucose deficiency in multicell spheroids: implications for tumor angiogenesis. *Proc Natl Acad Sci USA* **92**: 768–772

Takahashi Y, Kitadai Y, Bucana CD, Cleary KR and Ellis LM (1995) Expression of vascular endothelial growth factor and its receptor, KDR, correlates with vascularity, metastasis, and proliferation of human colon cancer. *Cancer Res* **55**: 3964–3968

Takahashi Y, Bucana CD, Cleary KR and Ellis LM (1998) p53, vessel count, and vascular endothelial growth factor expression in human colon cancer. *Int J Cancer* **79**: 34–38

© 2000 Cancer Research Campaign

Tufto I, Lyng H and Rofstad EK (1998) Vascular density in human melanoma xenografts: relationship to angiogenesis, perfusion and necrosis. *Cancer Lett* **123**: 159–165

Westphal JR, van't Hullenaar RG, van der Laak JA, Cornelissen IM, Schalkwijk LJ, van Muijen GN, Wesseling P, de Wilde PC, Ruiter DJ and de Waal RM (1997) Vascular density in melanoma xenografts correlates with vascular permeability factor expression but not with metastatic potential. *Br J Cancer* **76**: 561–570

© 2000 Cancer Research Campaign
